# Effect of praziquantel treatment of *Schistosoma mansoni *during pregnancy on immune responses to schistosome antigens among the offspring: results of a randomised, placebo-controlled trial

**DOI:** 10.1186/1471-2334-11-234

**Published:** 2011-09-02

**Authors:** Robert Tweyongyere, Patrice A Mawa, Macklyn Kihembo, Frances M Jones, Emily L Webb, Stephen Cose, David W Dunne, Birgitte J Vennervald, Alison M Elliott

**Affiliations:** 1 Faculty of Veterinary Medicine, Makerere University, Kampala, Uganda; 2 Medical Research Council/Uganda Virus Research Institute-Uganda Research Unit on AIDS, Entebbe, Uganda; 3 Department of Pathology University of Cambridge, Cambridge, UK; 4 London School of Hygiene and Tropical Medicine, London, UK; 5 DBL-Centre for Health Research and Development, Department of Veterinary Disease Biology, University of Copenhagen, Copenhagen, Denmark

## Abstract

**Background:**

Offspring of women with schistosomiasis may exhibit immune responsiveness to schistosomes due to *in utero *sensitisation or trans-placental transfer of antibodies. Praziquantel treatment during pregnancy boosts maternal immune responses to schistosome antigens and reduces worm burden. Effects of praziquantel treatment during pregnancy on responses among offspring are unknown.

**Methods:**

In a trial of anthelminthic treatment during pregnancy in Uganda (ISRCTN32849447; http://www.controlled-trials.com/ISRCTN32849447/elliott), offspring of women with *Schistosoma mansoni *were examined for cytokine and antibody responses to schistosome worm (SWA) and egg (SEA) antigen, in cord blood and at age one year. Relationships to maternal responses and pre-treatment infection intensities were examined, and responses were compared between the offspring of women who did, or did not receive praziquantel treatment during pregnancy.

**Results:**

Of 388 *S. mansoni-*infected women studied, samples were obtained at age one year from 215 of their infants. Stool examination for *S. mansoni *eggs was negative for all infants. Cord and infant samples were characterised by very low cytokine production in response to schistosome antigens with the exception of cord IL-10 responses, which were substantial. Cord and infant cytokine responses showed no association with maternal responses. As expected, cord blood levels of immunoglobulin (Ig) G to SWA and SEA were high and correlated with maternal antibodies. However, by age one year IgG levels had waned and were hardly detectable. Praziquantel treatment during pregnancy showed no effect on cytokine responses or antibodies levels to SWA or SEA either in cord blood or at age one year, except for IgG1 to SWA, which was elevated in infants of treated mothers, reflecting maternal levels. There was some evidence that maternal infection intensity was positively associated with cord blood IL-5 and IL-13 responses to SWA, and IL-5 responses to SEA, and that this association was modified by treatment with praziquantel.

**Conclusions:**

Despite strong effects on maternal infection intensity and maternal immune responses, praziquantel treatment of infected women during pregnancy had no effect on anti-schistosome immune responses among offspring by age one year. Whether the treatment will impact upon the offspring's responses on exposure to primary schistosome infection remains to be elucidated.

**Trial registration:**

ISRCTN: ISRCTN32849447

## Background

Protection against infection encountered in the first few weeks of childhood is thought to be mediated primarily by maternally-derived immunoglobulin [1,2]. However, it has been established that neonatal T cells are able to respond to specific antigens [3-5], and neonatal B cells are also capable of producing antibodies in response to specific antigens [6]. Additionally, infection during pregnancy [6-8], or antigen exposure through immunisation during pregnancy [9], may also lead to immunological sensitisation of the developing foetus to that particular infection or immunogen. For schistosome antigens, *in utero *sensitisation has been shown to occur in up to 50% of human neonates whose mothers had schistosomiasis [6,10]. Possible mechanisms for such sensitisation include direct exposure to antigens crossing the placenta [11-13] or transfer of idiotypic antibodies that cross the placenta [14,15]. Supporting the first hypothesis, Attallah and colleagues [13] described the presence of a 63-KD *S. mansoni *antigen in 86% of cord blood samples, suggesting that a high proportion of infants in schistosomiasis endemic populations are exposed to worm antigens *in utero*. Supporting the second hypothesis, studies in mice have demonstrated that *in utero *idiotypic exposure induces B and T cell responsiveness to schistosome antigens recognised by the idiotype [16], and that this neonatal exposure may be an important immmunoregulatory factor in subsequent schistosomiasis [17,18]. In addition, studies have reported that the effects of helminth infection during pregnancy on immune responses in neonates may also extend to non-helminth antigens [19,20].

It is estimated that approximately 40 million women of child bearing age are infected with schistosomiasis [21], and that the number of women with schistosomiasis during pregnancy in Africa could be as high as 10 million per year [22]. Previous policy has excluded pregnant and lactating women from the control of schistosomiasis using praziquantel treatment [23], but this policy was rescinded following a review in 2002 [22,24]. Despite this, there has been no information on the effects of praziquantel treatment of schistosomiasis during pregnancy on subsequent immune responses of the mothers' babies. We hypothesised that treatment of schistosomiasis during pregnancy might impact upon *in utero *sensitisation and thus the immune responsiveness of babies born to infected and treated women. We previously showed that praziquantel treatment during pregnancy boosted both cytokine responses [25] and antibody levels [26] to schistosome antigens in mothers six weeks after treatment. We now report findings from a study of offspring of women with *S. mansoni *infection during pregnancy. We show that immune responsiveness of the offspring seen in cord blood and in infants at age one year to schistosome antigens was lower than seen in their mothers. We also show that praziquantel treatment induced boosts in maternal immune responses to schistosome worm (SWA) and egg (SEA) antigens were still evident at the time of delivery, but there were no similar boost effects seen among their offspring.

## Methods

### Study setting, subjects and sample collection

This study was nested within the larger 'Entebbe Mother and Baby Study' (EMaBS), which was designed to investigate the impact of helminths and their treatment on the response to immunisation and on susceptibility to infectious diseases in childhood in Uganda (ISRCTN 32849447). As previously described [27], EMaBS was conducted within Entebbe municipality and the adjacent Katabi sub-county in Uganda. The study was a randomised, double-blind placebo-controlled trial of praziquantel versus matching placebo and albendazole versus matching placebo during pregnancy in a 2 × 2 factorial design. Women were recruited to the study between April 2003 and November 2005. At enrolment, a stool sample was obtained and examined for intestinal helminths including *S. mansoni *infection. Two slides from a single stool sample were examined by the Kato Katz method, as previously described [26], and infection intensity categorised as undetected, light (0-99 eggs per gram (epg)), moderate (100-399 epg) or heavy (> 400 epg). The randomised trial treatments were given after obtaining the stool sample, and during the second or third trimester of pregnancy. All women were treated with both albendazole and praziquantel approximately six weeks after delivery.

For the current study, we focussed on women infected with *S. mansoni *and their infants. Blood samples used in this analysis were obtained from women within one week following delivery, from cord blood, and from infants at age one year. Cord blood samples were obtained, after clamping, from the placental side of the cord using a needle and syringe. From November 2003, samples from women with *S. mansoni *infection and their offspring were examined for cytokine responses to schistosome worm (SWA) and egg (SEA) antigens in a whole blood assay. Stored plasma samples from *S. mansoni-*infected women and their offspring were examined for levels of antibodies to SWA and SEA antigens.

Possible contamination of cord blood with maternal blood at time of birth was assessed by measuring beta-human chorionic gonadotropin (_β_HCG) in cord blood serum by ELISA (Diagnostic Automation Inc. California USA). This hormone is found in high concentrations in maternal blood, but does not cross the placenta [28].

### Ethical consideration

Written informed consent was obtained from each participant as previously described [27]. For the infants' participation, consent was obtained from the mother or father or guardian if both parents were deceased. Ethical approval was obtained from the Science and Ethics Committee of Uganda Virus Research Institute - Ministry of Health, the Uganda National Council for Science and Technology and the London School of Hygiene and Tropical Medicine.

### Whole blood culture and cytokine assay

Whole blood culture stimulation for cytokine responses to SWA and SEA was performed as described elsewhere [25,29,30]. Briefly, heparinised blood was diluted to a final concentration of 1 in 4 with serum-free medium (RPMI supplemented with glutamine, penicillin and streptomycin) and 200 μl per well added to 96-well, round-bottomed plates (TC Microwell, NUNC A/S, Roskelde, Denmark). The blood was stimulated with SWA, SEA or phytohaemagglutinin (PHA - Sigma, UK) at final concentrations of 10 μg/ml, or was left unstimulated. Cultures were incubated at 37°C with 5% CO_2_, and supernatants harvested after 6 days. Supernatants were virally inactivated with 0.03% tributyl phosphate and 1% Tween 80 (Sigma) at room temperature for one hour and stored at -80°C. Maternal and cord blood samples were assessed for interferon gamma (IFN_γ_), IL-2, IL-4, IL-5, IL-13 and IL-10 responses whilst one year samples were assessed for IFN_γ_, IL-5, IL-13 and IL-10 responses. SWA and SEA were prepared in Professor David Dunne's laboratory, as previously described [31]. Levels of endotoxin in the antigens, measured using a Limulus Amebocyte Lysate Kit, QCL-1000 (BioWhittaker Inc, Walkersville, MD, USA) were 0.086 EU/mg of SWA and 0.175 EU/mg of SEA. At 10 μg/ml antigen concentration the endotoxin levels in culture were therefore negligible (< 0.1 ng/ml) and unlikely to cause any significant cytokine responses in whole blood stimulation.

IFNγ, IL-2, IL-4, IL-5 and IL-10 production in response to SWA and SEA stimulation of blood was measured in supernatants using OptEIA ELISA Kits (BD Pharmingen, USA). For IL-13, antibody pairs (BD PharMingen, USA), with standards from the National Institute for Biological Standards and Controls (NIBSC, UK) were used. The sensitivity of each assay, and cut-off for detectable responses, was the lowest concentration on the standard curve (7.8 pg/ml, except for IFNγ which was 8.6 pg/ml for maternal and cord responses and 26 pg/ml for age one year responses). Cytokine concentration in unstimulated wells was subtracted from concentrations in antigen-stimulated wells to obtain the antigen-specific response.

### Assay of antibodies against schistosome antigens

Levels of IgE, IgG1, IgG2, IgG3 and IgG4 against SWA and SEA were measured in plasma samples and in duplicate by ELISA as previously described [26]. Briefly, flat bottomed 96-well styrene microtitre-9205 plates (Thermo Labsystems, USA) were coated with SWA (8 μg/ml) or SEA (2.4 μg/ml) in 100 μl bicarbonate coating buffer and incubated overnight at 4°C. A standard positive pool was used for quantification of antibodies. Biotinylated mouse anti-human monoclonal antibodies were used for detection and were obtained from BD Pharmingen (San Diego USA), with the exception of IgG3 which was obtained from Zymed (S. San Francisco, USA). Poly-HRP-streptavidin conjugate (Sanquin, Netherlands) was added at a 1/4000 dilution. Plates were developed with OPD substrate and the reaction stopped by addition of 25 μl per well of 2 M sulphuric acid on observing the colour change at 15 minutes for IgG1-4 and 20 minutes for IgE. Optical densities (ODs) were recorded into data files using DeltaSOFT II (BioMetalics, Inc USA) and exported into Microsoft Excel and Stata (StataCorp, USA). Concentrations were calculated from ODs by interpolation from standard curves using Stata 5.0. The sensitivity of the test was determined as the lowest standard concentration above which levels were detectable (39 μg/ml, 0.7 μg/ml, 0.195 μg/ml, 0.32 μg/ml and 2.6 μg/ml for IgG1-4 and IgE to SWA respectively; 2 μg/ml, 0.2 μg/ml, 0.022 μg/ml, 0.1 μg/ml and 0.16 μg/ml for IgG1-4 and IgE to SEA respectively).

### Statistical analysis

The analysis had five objectives. Firstly, we examined whether the booster effects of treatment during pregnancy on the maternal response that we have previously observed were still apparent at time of delivery by comparing cytokine responses and antibody levels between women in the praziquantel and placebo groups. Secondly, we examined the effect of praziquantel treatment during pregnancy on the level of immune responsiveness of the offspring to schistosome antigens. This was done by comparing the levels of cytokine production in response to SWA and SEA in cord blood and at age one year between offspring of women of the praziquantel and placebo groups. Thirdly, we examined the effect of praziquantel treatment on maternal antibody transfer by comparing cord blood antibody levels between the praziquantel and placebo groups. Fourthly, we examined for associations between maternal immune responses and responses in their offspring by correlation analysis of maternal antibodies and cytokine responses with responses in cord blood and at age one year. Lastly, we examined the effect of maternal *S. mansoni *infection intensity on immune responsiveness to schistosome antigens among the women and their offspring by correlation analysis of infection intensity with cytokine responses and antibody levels.

Cytokine and antibody responses to *S. mansoni *antigens SWA and SEA showed skewed distributions, some with large numbers of undetectable results, which were set to zero. Responses were transformed using log_10_(concentration + 1), but many distributions remained skewed. For each cytokine and each antibody, the non-parametric Wilcoxon ranksum test was used on the log-transformed data to examine the effect of praziquantel treatment on the level of response. Binary variables representing detectable/non-detectable levels for each cytokine and antibody were also analysed; logistic regression was used to estimate odds ratios (ORs) and 95% CIs for the association between praziquantel treatment and having a detectable response.

Spearman's rank correlation coefficients were calculated to examine the relationship between maternal responses and those in cord blood and at one year. The relationship between *S. mansoni *infection intensity in the mother and level of cytokine and antibody response in cord blood, and at one year was also examined using Spearman's correlation. In order to examine whether the effect of praziquantel treatment on immune responses was modified by infection intensity, the Spearman's correlation coefficients for the relationship between infection intensity and cytokine response were compared between the treatment arms by applying Fisher's transformation to the correlation coefficients and conducting a z-test on these transformed values [32].

## Results

Three hundred and eighty-eight women had *S. mansoni *detected at enrolment and these constituted the nested cohort within the larger EMaBs cohort for this study; 201 were randomized to placebo and 187 to praziquantel treatment. Infection intensity at enrolment was previously reported [26]; 65% had light, 19% moderate and 16% heavy *S. mansoni *infection intensity. The median egg count was 36 epg (range 12 to 7026 epg). At delivery 125 women in the placebo group and 127 women in the praziquantel group provided blood samples. On average the time between treatment and when the delivery samples were drawn was 100 days (SD 48 days).

Cord blood _β_HCG levels did not differ between the placebo and praziquantel group. Cord blood samples with _β_HCG concentrations above 166 mIU/ml were considered as potentially contaminated with maternal blood and were therefore excluded from the analysis; 14 cord blood samples were excluded on this basis. Cord blood obtained from 110 babies in the placebo group and 102 in the praziquantel group were included. At age one year, 225 infants, 107 from the placebo group and 118 from the praziquantel group, provided blood and stool samples. None of these infants had eggs of *S. mansoni *detected in their stool.

In the whole blood assays, cytokine levels in unstimulated wells were generally low; medians (interquartile ranges) for maternal IFNγ, IL-2, IL-4, IL-5, IL-10 and IL-13 levels were 39.5(16.0, 67.0), 16 (0, 30.2), 0 (0, 15.0), 0 (0,11.0) 20.5 (8, 32.0) and 0 (0,9.3) pg/ml respectively. Cord blood levels were 0(0, 19.9), 0 (0, 13.8), 0(0,0), 0(0,0), 8(0, 27.1) and 0(0, 0) pg/ml respectively. Levels among infants were 0(0,59.5), 0 (0, 0), 13.0 (0, 34.0), 0(0,10.3) for IFNγ, IL-5, IL-10 and IL13 respectively. The levels did not differ between the placebo and praziquantel groups at any time point. Only results for net production in response to stimulation are shown hereafter.

### Maternal cytokine and antibody responses to SWA and SEA at delivery

Whole blood assay and antibody measurement were performed on maternal samples drawn from women within a week after delivery. Measurement of cytokine responses was performed on 164 maternal blood samples of which 75 (45.7%) were from women who received placebo during pregnancy while 89 (54.3%) were from women who received praziquantel. Specific antibody levels were measured in 252 samples drawn from women within a week after delivery; of these, 125 (49.6%) received placebo and 127 (50.4%) received praziquantel.

**Figure 1:**
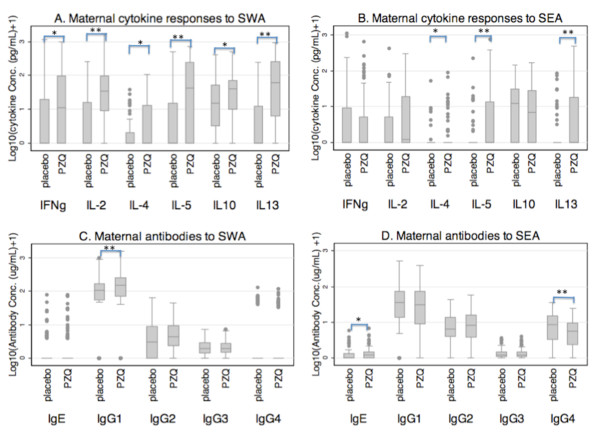
**Cytokine responses and antibody levels among women at delivery who received praziquantel treatment during pregnancy (PZQ) or who were left untreated (placebo)**. A and B show levels of cytokine production to SWA and SEA respectively plotted as log_10 _(cytokine concetration (pg/ml)+1). C and D show antibody levels to SWA and SEA respectively plotted as log10 (antibody concentration (in μg/mL)+1). Corresponding data on cytokine and antibody levels are given in additional file 1 and additional file 2 respectively. *p < 0.05; **p < 0.001.

At delivery, maternal cytokine responses to SWA stimulation (Figure 1A) were significantly higher for all cytokines tested in mothers that were treated compared to those that were not (p = 0.002 for IFNγ, IL-4 & IL-10; p < 0.001 for IL-2, IL-5 &IL-13). Similarly, in response to SEA stimulation (Figure 1B), production of IL-4, IL-5 and IL-13, but not IFNγ or IL-10, was significantly higher in the praziquantel treated group compared to placebo (p = 0.022 for IL-4 and < 0.001 for IL-5 & IL-13).

Maternal antibodies at delivery showed higher levels of SWA-specific IgE (p = 0.054), and IgG1 (p < 0.001) in treated mothers (Figure 1C). For SEA-specific antibodies (Figure 1D), IgE levels were higher in the treated mothers than in the placebo group (p = 0.048) while IgG4 levels were lower in treated mothers compared to the placebo group (p = 0.001).

Overall, these results show that praziquantel treatment in the second or third trimester induces an extended and significant boost, still detectable at delivery, in schistosome antigen-specific maternal cytokine responses, and in most antibody levels.

### Cord blood and infant cytokine responses to *S. mansoni *antigens

Whole blood assay and measurement of cytokine responses was performed on 144 samples of cord blood and 225 blood samples from infants at age one year. Of the 144 cord blood samples, 70 (48.6%) were from babies of women who received placebo during pregnancy and 74 (51.4%) were from babies of women who received praziquantel. Of the 225 samples taken at age one year, 107 (47.6%) were from babies whose mothers received placebo and 118 (52.4%) from babies of mothers who received praziquantel.

**Figure 2:**
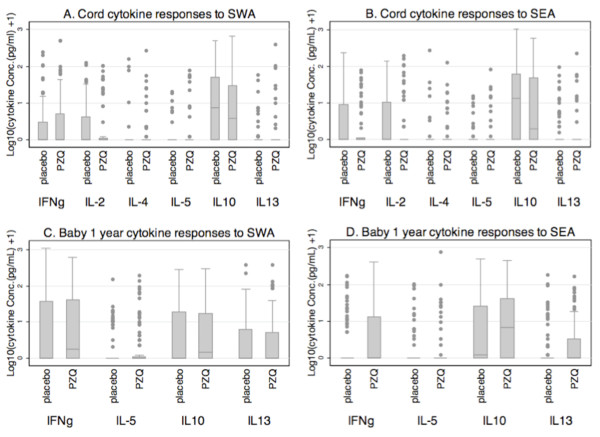
**Cytokine responses to SWA and SEA in cord blood (A and B) and infants at age one year (C and D)**. Box plots show levels of cytokine production plotted by maternal praziquantel treatment; that is, women who received praziquantel treatment during pregnancy (PZQ) or who were left untreated (placebo). Plotted on the y-axis is log_10 _(cytokine concentration (pg/mL) + 1). Corresponding data are given in additional file 1.

Cord blood cytokine responses to both SWA and SEA were generally low, the exception being noticeable amounts of IL-10 produced in response to both SWA and SEA antigens (Figures 2A and 2B, respectively). Cord blood production of IFNγ, IL-2, IL-4, IL-5, IL-13 and IL-10 in response to SWA stimulation was seen in 29.4%, 28.5%, 13.9%, 12.5%, 15.5% and 58.2% of the samples respectively, and the corresponding figures for responses to SEA were 32.2%, 32.9%, 13.9%, 13.9%, 18.3%, and 58.2% respectively. Overall 81.4% and 80.0% of the samples produced at least one of the cytokines in response to SWA and SEA respectively. On excluding IL-10 (in order to focus on cytokines more likely to be of lymphocyte origin), 55.3% and 62.4% of the cord samples produced at least one of the cytokines in response to SWA and SEA respectively.

The levels of cytokine responses to SWA and SEA in cord were mostly lower than in mothers at delivery (compare Figures 1A and 1B with Figures 2A and 2B; also additional file 1) and, for each cytokine, there was no significant association between the maternal and cord blood cytokine response. Comparing maternal treatment arms, praziquantel during pregnancy had no significant effect on any specific cytokine response in cord blood. That is, the differences between the placebo and praziquantel groups seen among maternal responses were not seen among the cord responses (Figure 2A and 2B). Thus, overall, praziquantel treatment during pregnancy appeared to have no effect on the cytokine response in cord blood.

However, there was a suggestion that the association between maternal infection intensity and cord blood responses differed depending on whether the mother received praziquantel or placebo. Cord blood IL-5 and IL-13 responses to SWA, and IL-5 responses to SEA, were positively correlated with maternal infection intensity at enrolment in the placebo group but not in the praziquantel group; IL-2 responses showed a similar trend (Table 1) although a formal test of differences between each pair of correlation coefficients found no strong evidence for a difference. Further exploration of the data seemed to suggest that the lack of association within the praziquantel group was explained by a tendency for praziquantel to induce increased cord blood responses among infants of mothers with low intensity infection and decreased responses among infants of mothers with moderate to heavy infection (Table 2).

**Table 1:** Spearman correlation between cord blood responses and maternal infection intensity at enrolment during pregnancy.

		Cytokine responses to SWA			Cytokine responses to SEA		
		Spearman coefficients	p-value	p-value for difference between correlations	Spearman coefficients	p-value	p-value for difference between correlations
IFNγ	Placebo (n = 70)	0.0426	0.726	0.45	0.1326	0.274	0.26
	Praziquantel (n = 73)	-0.0901	0.449		-0.0634	0.594	
IL-2	Placebo (n = 65)	0.2134	0.088	0.28	0.1468	0.243	0.21
	Praziquantel (n = 72)	0.0214	0.858		-0.0789	0.510	
IL-4	Placebo (n = 66)	-0.1362	0.275	0.64	0.0064	0.960	0.98
	Praziquantel (n = 71)	-0.2166	0.070		0.0111	0.927	
IL-5	Placebo (n = 70)	0.2744	0.021	0.09	0.2271	0.059	0.40
	Praziquantel (n = 74)	-0.0169	0.886		0.0830	0.482	
IL-13	Placebo (n = 69)	0.2644	0.028	0.09	0.1554	0.202	0.39
	Praziquantel (n = 73)	-0.0331	0.781		0.0044	0.971	
IL-10	Placebo (n = 69)	0.0178	0.885	0.77	-0.0384	0.754	0.92
	Praziquantel (n = 72)	0.0691	0.564		-0.0572	0.633	

**Table 2:** Cytokine responses (geometric mean concentration (pg/mL) +1) and antibody levels (geometric mean concentration (μg/mL) +1) of cord blood by infection intensity and by treatment

		For women with light infection^a^	For women with moderate to heavy infection^b^	For women with light infection^a ^	For women with moderate to heavy infection^b ^
**Cord blood cytokine responses to SWA**				**Cord blood cytokine responses to SEA**	

IFNγ	placebo	2.28 (1.47, 3.53)	2.36 (1.19, 4.69)	2.57 (1.65, 3.99)	3.39 (1.65, 6.97)
	praziquantel	3.18 (1.90, 5.34)	1.45 (1.01, 2.09)	1.84 (1.30, 2.60)	1.95 (1.08, 3.52)
IL-2	placebo	2.02 (1.40, 2.91)	3.07 (1.35, 6.96)	2.36 (1.57, 3.55)	4.65 (2.12, 10.2)
	praziquantel	1.97 (1.31, 2.95)	2.44 (1.23, 4.83)	2.39 (1.47, 3.89)	1.67 (1.05, 2.64)
IL-4	placebo	1.39 (0.97, 1.98)	1.12 (0.89, 1.41)	1.29 (0.96, 1.73)	1.35 (0.87, 2.08)
	praziquantel	1.79 (1.23, 2.60)	1.12 (0.94, 1.34)	1.53 (1.13, 2.08)	1.26 (0.90, 1.76)
IL-5	placebo	1.06 (0.97, 1.15)	1.73 (1.08, 2.77)	1.09 (1.00, 1.20)	1.57 (1.02, 2.40)
	praziquantel	1.53 (1.09, 2.14)	1.36 (0.95, 1.96)	1.30 (1.02, 1.67)	1.46 (0.99, 2.16)
IL-13	placebo	1.22 (0.99, 1.51)	1.68 (1.04, 2.72)	1.76 (1.24, 2.50)	1.60 (1.06, 2.40)
	praziquantel	1.49 (1.08, 2.05)	1.52 (0.84, 2.76)	1,40 (1.04, 1.87)	1.63 (0.92, 2.87)
IL-10	placebo	10.09 (5.38, 18.92)	6.72 (2.66, 17.04)	11.64 (5.95, 22.77)	10.32 (3.83, 27.8)
	praziquantel	5.91 (3.18, 10.99)	7.40 (3.18, 17.23)	7.08 (3.69, 13.58)	5.96 (2.39, 14.87)

**Cord antibodies against SWA**				**Cord antibodies against SEA**	

IgG1	placebo	39.87 (25.24, 62.99)	166.41 (104.02, 266.23)	53.94 (38.99, 74.63)	36.20 (21.16, 61.91)
	praziquantel	73.70 (44.18, 122.94)	317.85 (243.33, 415.19)	46.22 (31.35, 68.15)	29.22 (17.16, 49.77)
IgG2	placebo	2.14 (1.72, 2.68)	4.48 (3.03, 6.62)	8.33 (6.70, 10.35)	7.12 (5.38, 9.43)
	praziquantel	2.63 (2.04, 3.38)	5.80 (4.29, 7.84)	7.72 (5.99, 9.95)	6.49 (4.95, 8.52)
IgG3	placebo	1.79 (1.61, 2.00)	1.64 (1.45, 1.86)	1.28 (1.21, 1.36)	1.17 (1.10, 1.25)
	praziquantel	1.59 (1.46, 1.73)	2.10 (1.77, 2.49)	1.24 (1.18, 1.31)	1.36 (1.23, 1.51)
IgG4	placebo	1.11 (0.96, 1.28)	2.24 (1.27, 3.97)	4.07 (3.24, 5.11)	7.32 (5.04, 10.6)
	praziquantel	1.27 (1.01, 1.60)	2.96 (1.55, 5.65)	3.42 (2.71, 4.32)	7.61 (5.71, 10.16)

^a ^for cytokines n = 45-48 in placebo group and 49-51 in praziquantel group; for antibodies n = 73 placebo group and n = 67 in praziquantel group

^b ^for cytokine responses n = 20 - 22 in placebo group and 23 in praziquantel group; for antibodies n = 37 in placebo group and n = 35 in praziquantel group

Responses in infants at one year still showed low levels of cytokine production in response to both SWA and SEA (Figure 2C and 2D, respectively). There was no difference in the responses in infants at one year between those that were born of mothers who were treated with praziquantel, and those that were not. For each cytokine, infants' responses were not associated with cord responses.

### Anti-schistosome antibody levels in cord blood and infants at age one year

Antibody levels were measured in 212 cord blood samples and 127 blood samples from infants at age one year. Of the 212 cord blood samples, 110 (51.9%) were from the placebo group and 102 (48.1%) from the praziquantel group. Of the 127 infant samples 62 (48.8%) were offspring of mothers who received placebo and 65 (51.2%) were offspring of mothers who received praziquantel.

**Figure 3:**
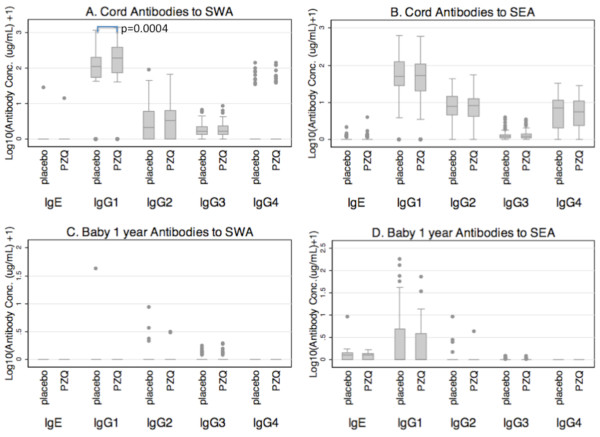
**Antibodies against SWA and SEA in cord blood (A and B) and infants at age one year (C and D)**. Box plots show levels of each antibody by maternal treatment; that is, women who received praziquantel treatment during pregnancy (PZQ) or who were left untreated (placebo). Plotted on the y-axis is log_10 _(antibody concentration (μg/mL) + 1). Corresponding data are given in additional file 2. *p = 0.0004.

Overall, the pattern of IgG isotypes to schistosome antigens in cord blood was similar to that seen in mothers at delivery, regardless of their treatment group: high levels of IgG1 and low levels of IgG2 and IgG3 to SWA (compare Figure 1C with Figure 3A); high levels of IgG1 and low levels of IgG2, IgG3 and IgG4 to SEA (compare Figure 1D with Figure 3B). On the other hand, IgE to SWA and SEA was detectable in very few cord blood samples (2/212 and 34/212 respectively).

**Figure 4:**
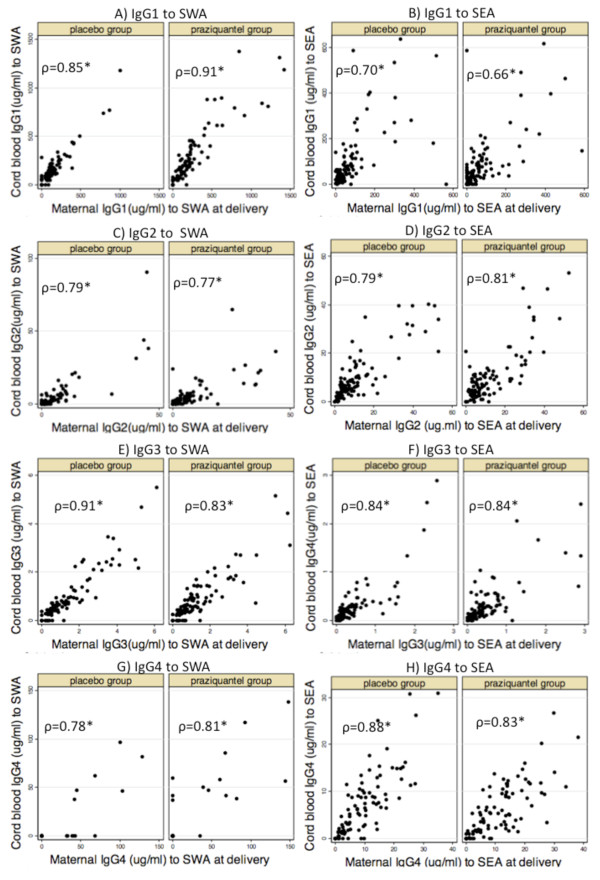
**Correlations between maternal and cord blood levels of antibody to SWA and SEA**. *Spearman's correlation coefficients; p < 0.001.

As expected [33], cord blood levels of IgG to SWA and SEA were correlated with maternal levels in both treatment groups with Spearman's coefficients ranging from 0.66-0.91, (p < 0.001) for all IgG isotypes (Figure 4), although there were some subtle differences, in keeping with recognised differences in transport across the placenta [34]. For example, in both the placebo and praziquantel groups, maternal levels of IgG3 to SWA were higher than cord blood levels and maternal levels of IgG1 to SEA were lower than cord blood levels (additional file 2). Paralleling maternal responses, treatment with praziquantel was associated with increased cord blood antibody levels to SWA; this was statistically significant for IgG1 (also see Figure 3A).

Levels of maternal and cord blood IgG1, IgG2 and IgG4 against SWA and IgG4 against SEA were positively correlated with maternal enrolment *S. mansoni *infection intensity while levels of IgG1, IgG2 and IgG3 against SEA showed a trend of negative correlation with maternal *S. mansoni *infection intensity (Table 3). Findings in cord blood mirrored those in maternal blood.

**Table 3:** Association between maternal or cord blood antibody levels and maternal infection intensity at enrolment

		Maternal Antibodies to SWA	Maternal Antibodies to SEA	Cord blood antibodies to SWA	Cord blood antibodies to SEA
IgG1	Placebo	0.45 (< 0.001)	-0.09 (0.314)	0.51 (< 0.001)	-0.15 (0.105)
	Praziquantel	0.53 (< 0.001)	-0.06 (0.517)	0.53 (< 0.001)	-0.18 (0.062)
IgG2	Placebo	0.22 (0.013)	-0.21 (0.019)	0.32 (< 0.001)	-0.20 (0.039)
	Praziquantel	0.39 (< 0.001)	-0.15 (0.099	0.45 (< 0.001)	-0.13 (0.192)
IgG3	Placebo	-0.10 (0.244)	-0.24 (0.008)	-0.10 (0.31)	-0.33 (< 0.001)
	Praziquantel	0.43 (< 0.001)	0.06 (0.532)	0.33 (< 0.001)	0.04 (0.674)
IgG4	Placebo	0.24 (0.008)	0.40 (< 0.001)	0.23 (0.016)	0.35 (< 0.001)
		0.31 (0.0003)	0.38 (< 0.001)	0.23 (0.019)	0.44 (< 0.001)
IgE	Placebo	0.29 (0.0009)	-0.17 (0.062)		0.02 (0.798)
	Praziquantel	0.21 (0.017)	-0.02 (0.855)		-0.03 (0.732)

Shown are Spearman's correlation coefficients (p-values); For maternal antibodies N = 125 in placebo group and 127 in praziquantel group. For cord blood antibodies N = 110 in placebo group and 102 in the praziquantel group.

Among the infants at age one year, there were hardly any detectable antibodies against SWA (Figure 3C); IgG1, IgG2 and IgG3 were detectable in 1/127, 6/127 and 19/127 infants respectively. No infant had detectable levels of IgG4 and IgE to SWA. In contrast to what was seen for antibodies to SWA, several infants had detectable IgG and IgE to SEA (Figure 3D); IgG1, IgG2 IgG3 and IgE to SEA were detectable in 42/127, 5/127, 18/127 and 89/127 infants respectively. These antibodies among infants were not associated with maternal or cord blood antibodies. Praziquantel treatment during pregnancy showed no significant effects on antibodies to SEA among infants.

## Discussion

This study describes the effect of *S. mansoni *infection and of praziquantel treatment during pregnancy on immune responsiveness to schistosome adult worm (SWA) and egg (SEA) antigens in the offspring of the affected women, examined in cord blood and in infants at age one year.

Our results show that the praziquantel-induced boost in worm-specific immune responses that was previously observed at six weeks after treatment [25,26] lasts at least up to the time of delivery. However, there was no evidence of a direct correlation between maternal and cord, or maternal and infant immune responses to schistosome antigens, except for the expected relationships between maternal and cord blood antibody levels.

In the current study IFNγ, IL-2, IL-4, IL-5, and IL-13 production in response to SWA or SEA stimulation was seen (for each cytokine) in approximately 10 to 30% of cord blood samples. Several earlier studies have also shown that uninfected children of infected mothers can have immune responses specific to *S. mansoni *[35-37] or *S. haematobium *[10]. Although these studies differed from ours in the *in vitro *assaying protocols used (using peripheral blood mononuclear cells rather than whole blood, or proliferation rather than cytokine assays), the findings nevertheless corroborate each other and suggest that a significant proportion of infants of infected women acquire T cell sensitisation *in utero*. Apart from IL-10, the levels of response observed in cord blood were much lower than those that were found among the mothers during pregnancy [25], or reported in infected children and adults living in schistosomiasis endemic areas [38-40]. Low T-cell responses in cord blood are expected since immune cells in cord blood are functionally immature [41-44].

However, an important finding was that the observed level and frequency of IL-10 response to schistosome antigens in cord blood was higher than that for other measured cytokine responses, and dominated the cord blood response profile. High IL-10 production is in line with the generally down-regulated responses of cord blood cells, although non-IL-10-mediated suppression may also be important in the fetal and neonatal response to antigens [45,46]. Because we were measuring whole blood culture supernatants, we have no information on the cellular sources of this IL-10. Possible sources include cells participating in the innate response, as well as T and B cells. One particular possibility may be fetal lineage regulatory T cells, which have been demonstrated to as biased towards immune tolerance [47,48]. If antigen-specific memory T or B cells are involved, this IL-10 production may reflect the acquisition of tolerance to schistosome antigens resulting from exposure in utero, with long-term implications for the response of such children on exposure to infection. The mechanisms of this effect deserve further investigation.

Given the measured effects of praziquantel during pregnancy on the prevalence and intensity of infection among mothers in this cohort (reduced by 81.9% [26]), and therefore, presumably, on levels of schistosome antigen in the maternal circulation, as well as the measured effects on maternal antibody and cytokine responses, we were surprised to observe no overall effect of praziquantel during pregnancy on *in utero *sensitisation, as assessed by cord blood responses to schistosome antigens. Several potential mechanisms may relate to this. One possible explanation is that sensitisation occurred prior to the provision of the intervention - equally in both groups. A second possible explanation is that the fetal and neonatal immune system itself has a default pathway towards tolerance [48] and thus the induction of measured antigen-specific responses (other than IL-10) was minimised - again equally in both groups. Alternatively, the possible interactions between maternal infection intensity and praziquantel may be important. Our data suggests a weak increase in sensitisation to worm antigens with increasing maternal infection intensity, especially for IL-5 and IL-13, which occurred in the placebo group, but not in the praziquantel group (although, the formal test for effect modification between the two groups was not significant). A plausible mechanism by which praziquantel might so modify an effect of maternal infection intensity may be as follows. Among infants of mothers with light infections, praziquantel may increase sensitisation by causing the release of antigen from dying worms into the maternal circulation and/or by boosting levels of anti-schistosome antibodies crossing the placenta. On the other hand, among infants of mothers with moderate to heavy infections, praziquantel treatment might reduce sensitisation by reducing the duration of fetal exposure to schistosome antigens. These suggestions are speculative and must be considered with caution given the weak evidence for these effects. Overall, our results do not support the hypothesis that praziquantel treatment during pregnancy influences sensitisation of the fetus to schistosome antigens.

Levels of IgG antibodies against SWA and SEA in cord blood were high and their correlation with maternal antibodies was expected since IgG antibodies cross the placenta [33]. Cord blood IgG1 to SWA, in particular, was increased as a result of maternal treatment with praziquantel. Antibodies acquired by trans-placental transfer are known to provide the major protection against bacterial and viral infections in early life [49]. Some protection against schistosome infection has been attributed to high levels of worm-specfic IgE [50,51], which was conspicuously absent in cord blood, however, maternal antibodies against schistosome antigens might modify the offspring's response to infection. In our setting, any such effect may not have been observed, as any infection that might have occurred during the first year of life was below detectable levels and antibody of maternal origin had waned by age one year. None of the infants in our study were found to be excreting *S. mansoni *eggs at detection sensitivity based on examination of a single stool sample. However, the low cytokine responses and low levels of IgE and IgG1 to SEA observed in several infants may actually be indicative of some low level early-stage infection not detected by single stool examinations. If so, this would corroborate a recent epidemiological study [52] that showed antibodies to SEA detectable in preschool children and infants as young as nine months old. Although the level of cytokine response to schistosome antigens in one-year olds was very low and no relationship between cord and infant responses was found, it will be important to determine whether or not *in utero *sensitisation does influence the subsequent response to schistosome antigens when children in the cohort do became detectably infected.

## Conclusions

This study found that although praziquantel treatment during pregnancy caused a boost in maternal immune responses to schistosome antigens that persisted up to the time of delivery, it had no overall influence on the immune responsiveness of uninfected offspring to schistosome antigens at age one year. There was a suggestion of an interaction between maternal infection intensity and the effect of praziquantel on *in utero *sensitisation to schistosome antigens. It will be of interest to determine whether praziquantel treatment during pregnancy impacts on the infant response when primary infection is encountered. This remains a question to be explored.

## Competing interests

The authors declare that they have no competing interests.

## Authors' contributions

RT participated in designing the study, carried out the immunoassays, did the data analysis, drafted the manuscript and coordinated writing the manuscript. PMA participated in the immunoassays. MK participated in the parasitological analysis of samples. FMJ participated in the immunoassays. ELW participated in data analysis and writing the manuscript. SC participated in writing the manuscript. DWD participated in designing the study and writing the manuscript. BJV participated in designing the study and writing the manuscript. AME conceived the study, participated in designing the study, data analysis and writing of the manuscript. All the authors read and approved the final manuscript.

## Pre-publication history

The pre-publication history for this paper can be accessed here:

http://www.biomedcentral.com/1471-2334/11/234/prepub

## Supplementary Material

Additional file 1 **Levels of cytokine response to schistosome worm antigen (SWA) and schistosome egg antigen (SEA) in maternal blood at delivery, cord blood and infant blood at age one year, by praziquantel versus placebo treatment during pregnancy**. A PDF file of a table showing levels of cytokine production following stimulation of either maternal cord or infant blood with SWA or SEA. Shown are median cytokine concentrations in pg/mL in whole blood culture supernatants. Click here for file

Additional file 2 **Levels of antibodies to schistosome worm antigen (SWA) and schistosome egg antigen (SEA) in maternal blood at delivery, cord blood and infant blood at age one year, by praziquantel versus placebo treatment during pregnancy**. A PDF file of a table showing the median plasma concentrations in μg/mL of antibodies to SWA and SEA and P-values for the ranksum comparison between maternal and cord blood levels. Click here for file
