# Supplementary material for: Effect of praziquantel treatment of Schistosoma mansoni during pregnancy on immune responses to schistosome antigens among the offspring: results of a randomised, placebo-controlled trial
Source: BMC Infect Dis. 2011 Sep 2;11:234. doi: 10.1186/1471-2334-11-234 (PMC3176493; doi:10.1186/1471-2334-11-234)
Supplement: Additional file 1 — Levels of cytokine response to schistosome worm antigen (SWA) and schistosome egg antigen (SEA) in maternal blood at delivery, cord blood and infant blood at age one year, by praziquantel versus placebo treatment during pregnancy. A PDF file of a table showing levels of cytokine production following stimulation of either maternal cord or infant blood with SWA or SEA. Shown are median cytokine concentrations in pg/mL in whole blood culture supernatants. [file 1471-2334-11-234-S1.PDF]

Additional file 1. Levels of cytokine response to schistosome worm (SWA) and egg (SEA) antigen for maternal responses at delivery, cord blood and infant responses at age one year, by praziquantel versus placebo treatment during pregnancy

	**Cytokine responses to SWA** Median cytokine concentration (IQR) in pg/ml.			**Cytokine responses to SEA** Geometric mean of cytokine concentration+1 (95% CI) in pg/ml.		
	**Maternal responses at delivery**	**Cord blood responses***	**Responses at age 1year**	**Maternal responses at delivery**	**Cord blood responses***	**Responses at age 1year**
**IFNγ**						
**Placebo group**	0 (0, 18.3)	0 (0, 2.3)	0 (0, 8.5)	0 (0, 8.0)	0 (0, 8.0)	0 (0, 0)
**Praziquantel group**	11.6 (0, 97.2)	0 (0, 4.5)	1 (0, 25.7)	0 (0, 5.4)	0 (0, 0.1)	0 (0, 12.5)
**IL2**						
**Placebo group**	0 (0, 14.7)	0 (0, 4.2)		0 (0, 3.8)	0 (0, 9.6)	
**Praziquantel group**	32.2 (6.5, 93.7)	0 (0, 0.2)		0 (0, 17.6)	0 (0, 0)	
**IL4**						
**Placebo group**	0 (0, 1)	0 (0, 0)		0 (0, 0)	0 (0, 0)	
**Praziquantel group**	0 (0, 12.2)	0 (0, 0)		0 (0, 0)	0 (0, 0)	
**IL5**						
**Placebo group**	0 (0, 14.4)	0 (0, 0)	0 (0, 0)	0 (0, 0)	0 (0, 0)	0 (0, 0)
**Praziquantel group**	46.7 (0, 229.9)	0 (0, 0)	0 (0, 0.1)	0 (0, 9.2)	0 (0, 0)	0 (0, 0)
**IL13**						
**Placebo group**	0 (0, 11.1)	0 (0, 0)	0 (0, 5.6)	0 (0, 0)	0 (0, 0.3)	0 (0, 0)
**Praziquantel group**	59.0 (4.7, 264.2)	0 (0, 0)	0 (0, 4.2)	0 (0, 15.8)	0 (0, 0)	0 (0, 2.2)
**IL10**						
**Placebo group**	14.0 (2.0, 51.0)	6.2 (0, 59.0)	0 (0, 18)	8 (0, 29.2)	12.0 (0, 64.1)	0.2 (0, 26.1)
**Praziquantel group**	39.0 (9.5, 80.9)	2.6 (0, 29.3)	0.6 (0, 16.5)	7 (0, 31.2)	1.2 (0, 46.0)	5.7(0, 41.6)
