# Supplementary material for: Effect of praziquantel treatment of Schistosoma mansoni during pregnancy on immune responses to schistosome antigens among the offspring: results of a randomised, placebo-controlled trial
Source: BMC Infect Dis. 2011 Sep 2;11:234. doi: 10.1186/1471-2334-11-234 (PMC3176493; doi:10.1186/1471-2334-11-234)
Supplement: Additional file 2 — Levels of antibodies to schistosome worm antigen (SWA) and schistosome egg antigen (SEA) in maternal blood at delivery, cord blood and infant blood at age one year, by praziquantel versus placebo treatment during pregnancy. A PDF file of a table showing the median plasma concentrations in μg/mL of antibodies to SWA and SEA and P-values for the ranksum comparison between maternal and cord blood levels. [file 1471-2334-11-234-S2.PDF]

Additional file 2. Levels of antibodies to schistosome worm (SWA) and egg (SEA) antigen in maternal blood at delivery, cord blood and infant blood at age one year, by praziquantel versus placebo treatment during pregnancy

	**Levels of antibodies to SWA** Median (IQR) in µg/ml.			**Levels of antibodies to SEA** Median (IQR) in µg/ml.		
	**Maternal levels at delivery**	**Cord blood levels**	**P-value***	**Maternal levels at delivery**	**Cord blood responses***	**P-value***
**IgG1**						
**Placebo group**	104.7 (81.3, 116.8)	106.2 (52.0, 202.5)	0.213	34.4(14.0, 72.7)	49.6 (26.2, 121.2)	0.01
**Praziquantel group**	165.0 (131.8, 201.3)	190.6 (72.8, 391.2)	0.210	29.0 (8.0, 69.5)	52.1 (19.5, 105.9)	0.003
**IgG2**						
**Placebo group**	2.2 (0, 7.3)	1.1 (0, 5.1)	0.088	5.3 (3.0, 12.7)	6.7 (3.6, 13.5)	0.332
**Praziquantel group**	3.3 (1.3, 8.1)	2.3 (0, 5.4)	0.044	6.7 (3.1, 14.4)	7.3 (3.1, 11.8)	0.965
**IgG3**						
**Placebo group**	1.0 (0.4, 2.0)	0.6 (0.3, 1.2)	0.019	0.2 (0.1, 0.5)	0.2 (0.1, 0.3)	0.443
**Praziquantel group**	1.0 (0.5, 1.8)	0.6 (0.3, 1.3)	0.002	0.2 (0.1, 0.4)	0.2 (0.1, 0.4)	0.381
**IgG4**						
**Placebo group**	0 (0, 0)	0 (0, 0)	0.292	7.7 (2.2, 14.2)	6.0 (1.0, 10.5)	0.091
**Praziquantel group**	0 (0, 0)	0 (0, 0)	0.518	4.5 (1.3, 9.2)	4.4 (1.2, 9.7)	0.691
**IgE**						
**Placebo group**	0 (0, 0)			0 (0, 0.3)	0 (0, 0)	
**Praziquantel group**	0 (0, 2.9)			0.2 (0, 0.4)	0 (0, 0)	

* ranksum comparison between maternal and cord blood antibody levels
